# Coronary Artery Disease–Associated *LIPA* Coding Variant rs1051338 Reduces Lysosomal Acid Lipase Levels and Activity in Lysosomes

**DOI:** 10.1161/ATVBAHA.116.308734

**Published:** 2017-05-24

**Authors:** Gavin E. Morris, Peter S. Braund, Jasbir S. Moore, Nilesh J. Samani, Veryan Codd, Tom R. Webb

**Affiliations:** From the Department of Cardiovascular Sciences, University of Leicester and National Institute for Health Research Leicester Cardiovascular Biomedical Research Unit, Glenfield Hospital, United Kingdom.

**Keywords:** coronary artery disease, genome-wide association study, lipase, macrophage

## Abstract

**Objective—:**

Genome-wide association studies have linked variants at chromosome 10q23 with increased coronary artery disease risk. The disease-associated variants fall in *LIPA*, which encodes lysosomal acid lipase (LAL), the enzyme responsible for lysosomal cholesteryl ester hydrolysis. Loss-of-function mutations in *LIPA* result in accelerated atherosclerosis. Surprisingly, the coronary artery disease variants are associated with increased *LIPA* expression in some cell types. In this study, we address this apparent contradiction.

**Approach and Results—:**

We investigated a coding variant rs1051338, which is in high linkage disequilibrium (*r*^2^=0.89) with the genome-wide association study lead–associated variant rs2246833 and causes a nonsynonymous threonine to proline change within the signal peptide of LAL. Transfection of allele-specific expression constructs showed that the risk allele results in reduced lysosomal LAL protein (*P*=0.004) and activity (*P*=0.005). Investigation of LAL localization and turnover showed the risk LAL protein is degraded more quickly. This mechanism was confirmed in disease-relevant macrophages from individuals homozygous for either the nonrisk or risk allele. There was no difference in LAL protein or activity in whole macrophage extracts; however, we found reduced LAL protein (*P*=0.02) and activity (*P*=0.026) with the risk genotype in lysosomal extracts, suggesting that the risk genotype affects lysosomal LAL activity. Inhibition of the proteasome resulted in equal amounts of lysosomal LAL protein in risk and nonrisk macrophages.

**Conclusions—:**

Our findings show that the coronary artery disease–associated coding variant rs1051338 causes reduced lysosomal LAL protein and activity because of increased LAL degradation, providing a plausible causal mechanism of increased coronary artery disease risk.

## Abstract 2

Supplemental Digital Content is available in the text.

Coronary artery disease (CAD), the most common cause of death worldwide,^1^ is caused by the build-up of atherosclerotic plaques within the artery wall. Plaques develop from endothelial damage, with subsequent inflammatory cell infiltration from circulating blood and migration/proliferation of surrounding structural cells, including medial vascular smooth muscle cells into the vessel’s intima. Initial extracellular lipid deposition occurs in the deep intima prior to macrophage recruitment.^2,3^ Subendothelial modified lipids are ingested predominantly by macrophages and vascular smooth muscle cells in an unregulated process, leading to excessive cholesteryl ester (CE) and triglyceride accumulation. Excess lipid accumulation in macrophages in the form of lipid droplets leads to differentiation into foam cells, which are abundant within atherosclerotic plaques, although new evidence suggests that a significant number of foam cells may be smooth muscle derived.^4^ Foam cells secrete proinflammatory cytokines and growth factors causing additional macrophage recruitment and matrix deposition, contributing to atherosclerotic lesion development.^5–7^

**See accompanying editorial on page 1015**

Genome-wide association studies have identified variants at chromosome 10q23, which associate with CAD.^8–11^ The lead variant, rs2246833, is associated with an ≈9% increased risk per copy of the CAD-associated allele. This variant and those in high linkage disequilibrium (LD) with it lie within or in close proximity to *LIPA*, which encodes lysosomal acid lipase (LAL).^12^ LAL is the key lysosomal enzyme for hydrolyzing CEs and triglycerides into free cholesterol (FC), glycerol, and free fatty acids and prevents lipid accumulation in various tissues and cell types, including macrophages.^13^

Loss-of-function mutations in *LIPA* cause either the rare autosomal recessive LAL deficiencies Wolman disease, where individuals possess negligible or no LAL activity, or CE storage disease, where patients retain some residual LAL activity. Both conditions are associated with extensive lysosomal CE and triglyceride accumulation in multiple tissues,^14^ and CE storage disease subjects develop premature atherosclerosis.^15–21^ Injection of recombinant LAL in low-density lipoprotein (LDL) receptor–deficient atherosclerotic mice prevented early atheroma formation and reduced the number of late-stage lesions,^22^ while injections of recombinant LAL into *LAL*-deficient mice reduced cholesterol and triglyceride levels in multiple tissues.^23–26^

As loss-of-function mutations in *LIPA* result in increased atherosclerosis in humans and overexpression of *LIPA* reduces atherosclerosis in mouse, the common CAD-associated variants in *LIPA* might be expected to cause a reduction in the levels of active LAL. Instead, the risk genotype has been found to be associated with increased *LIPA* gene expression in several tissues and cell types, including whole blood, peripheral blood mononuclear cells, monocytes, liver, and adipose.^27–30^ Here, we present evidence that addresses this apparent contradiction and suggest that a coding single nucleotide polymorphism in the signal peptide of LAL, which is in high LD with the CAD lead variant, affects LAL trafficking to the lysosome and is the likely causal variant at this locus.

## Materials and Methods

Materials and Methods are provided in the online-only Data Supplement.

## Results

### Coding Variant rs1051338 Is Predicted to Alter Processing of LAL Signal Peptide

**Figure 1.**
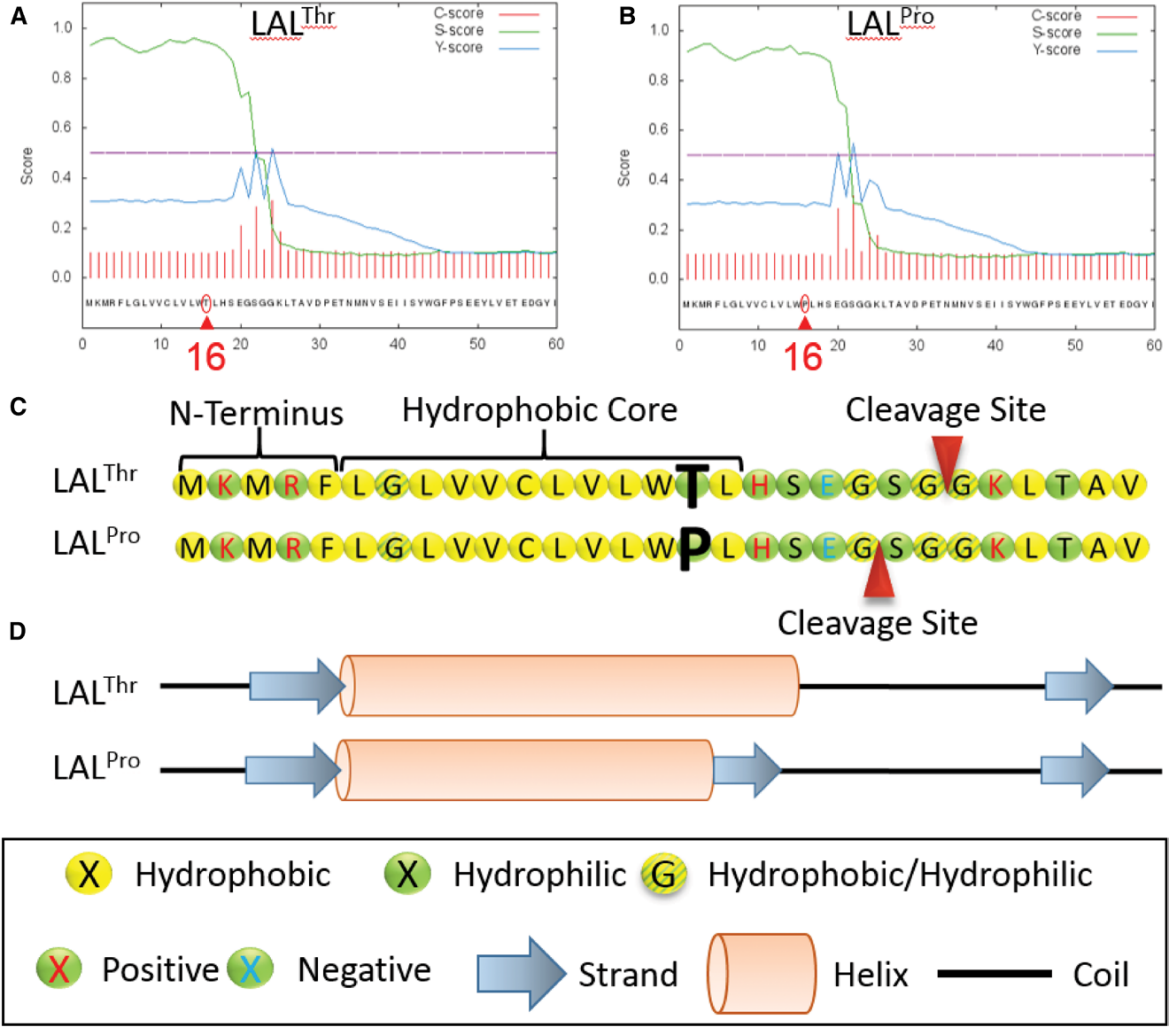
The predicted effect of p.Thr16Pro on the signal peptide function of lysosomal acid lipase (LAL). The signal peptide cleavage site was predicted using SignalP 4.1. The nonrisk coronary artery disease (CAD)–associated *LIPA* coding variant (LAL^Thr^; **A**) shows a predicted cleavage between position 23 and 24, and the risk CAD-associated *LIPA* coding variant (LAL^Pro^; **B**) shows a predicted cleavage between position 21 and 22. **C**, The likely positioning of the typical signal peptide 3-region composition: (1) hydrophilic positively charged N-terminus region, (2) the hydrophobic core region, and (3) the polar uncharged C-terminal (cleavage site) region recognized by signal peptidase. **D**, The secondary structural forms for the signal peptide predicted using PSIPRED.

Examination of rs2246833, the CAD-associated *LIPA* variant from genome-wide association studies, indicated a total of 13 proxy single nucleotide polymorphisms in high LD (*r*^2^>0.8; Table I in the online-only Data Supplement). One variant, rs1051338 minor allele frequency of 34%), causes a nonsynonymous threonine (LAL^Thr^, nonrisk) to proline (LAL^Pro^, risk) change at amino acid position 16 in the signal peptide of LAL. Signal peptides are found in the N-terminus of certain newly synthesized proteins and are responsible for targeting proteins for secretion or to specific organelles. Signal peptides comprise a hydrophilic N-terminus, a central α-helical hydrophobic core, which is required for cotranslational translocation from the cytosol to the endoplasmic reticulum, and a C-terminal cleavage site. We used in silico analyses tools to investigate how the threonine to proline amino acid change might affect LAL signal peptide processing. SignalP 4.1^31^ predicted that the signal peptide would be reduced by 2 amino acids in LAL^Pro^ compared with that in LAL^Thr^ (Figure 1A through 1C). PSIPRED 3.3^32,33^ predicted the LAL^Thr^ residue to contribute to an α-helix length of 13 residues, whereas the LAL^Pro^ residue was predicted to truncate the α-helix to 11 residues and introduce a 2 residue β-strand conformation at the carboxyl end of the α-helix, disrupting the hydrophobic core (Figure 1D).

### Effect of rs1051338 on LAL Localization

**Figure 2.**
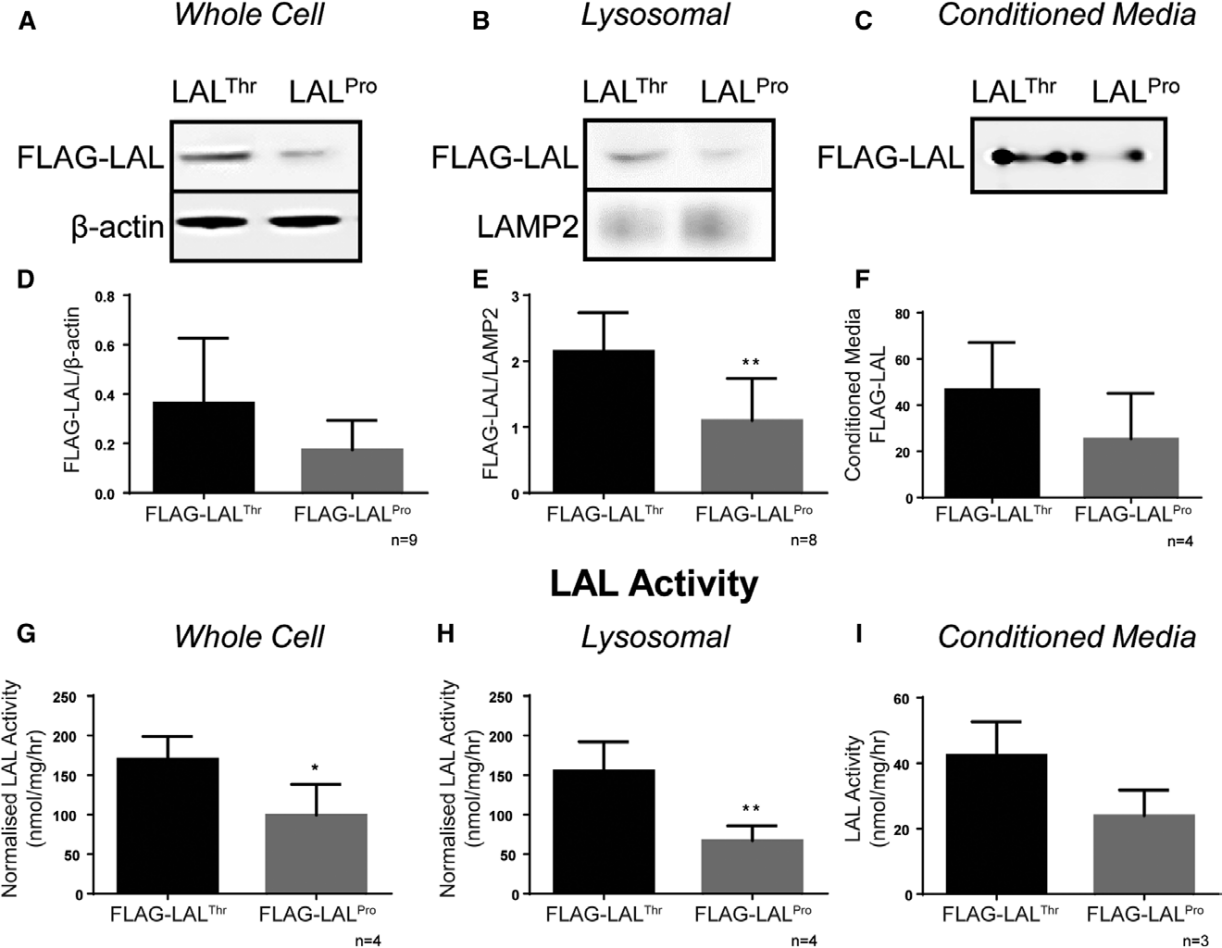
Risk coronary artery disease (CAD)–associated *LIPA* coding variant (LAL^Pro^) shows reduced expression and activity compared with nonrisk CAD-associated *LIPA* coding variant (LAL^Thr^). 1×10^6^ COS7 cells were transfected with 8 μg LAL-FLAG containing either LAL^Pro^ or LAL^Thr^ and 2 μg eGFP and cultured for 48 hours before whole cell, lysosomal lysates, and conditioned media were collected for LAL protein expression and activity analysis. Whole cell, lysosomal lysates, and conditioned media were immunoblotted for FLAG, with whole cell and lysosomal lysate levels normalized to β-actin or LAMP2 (lysosome-associated membrane protein 2), respectively. **A**–**C**, Representative whole cell, lysosomal, and conditioned media blots, with cumulative data in **D**–**F**, respectively. LAL activity in whole cell, lysosomal, and conditioned media fractions (**G**–**I**, respectively) were measured using the 4-MUP (4-methylumbelliferyl phosphate) assay and normalized to transfection efficiency and protein loading control. Data shown are mean±SD, with statistical significance indicated as *P<0.05 and **P<0.01, unpaired *t* test. LAL indicates lysosomal acid lipase.

To investigate the functional effects of, rs1051338 we generated allele-specific C-terminal FLAG-tagged LAL expression constructs. COS7 cells were transfected with either FLAG-LAL^Pro^ or FLAG-LAL^Thr^ 48 hours prior to sample collection and analysis. Because LAL is only functional in lysosomes, we enriched the cellular lysosomal fraction by differential centrifugation (Figure I in the online-only Data Supplement) and measured LAL protein expression and activity in whole cell, lysosomal, and conditioned media fractions from the same cell preparations. LAL protein levels were assessed using an anti-FLAG antibody and found to be borderline reduced within LAL^Pro^ whole cell extracts when compared with LAL^Thr^ (*P*=0.066; Figure 2A and 2D) and significantly reduced within the lysosomal fraction of LAL^Pro^ compared with LAL^Thr^ (*P*=0.004; Figure 2B and 2E), resulting in significantly reduced LAL activity within the whole cell (*P*=0.027; Figure 2G) and lysosomal fraction (*P*=0.005; Figure 2H). LAL^Pro^ protein levels (*P*=0.184; Figure 2C and 2F) and activity (*P*=0.069; Figure 2I) in the conditioned media of transfected cells also showed trends toward lower levels compared with LAL^Thr^, mirroring the trend seen intracellularly and suggesting a global reduction in LAL protein levels in LAL^Pro^ transfected cells. Of note, the difference in LAL protein levels was not caused by construct-specific transfection efficiency; there were equivalent levels of cotransfected eGFP protein expression in both conditions after 48 hours (*P*=0.858; Figure IIA and IIB in the online-only Data Supplement), and relative mRNA expression of each construct was equivalent after 24 hours (*P*=0.519; Figure IIC in the online-only Data Supplement).

### Coding Variant rs1051338 Causes Increased Degradation of LAL

**Figure 3.**
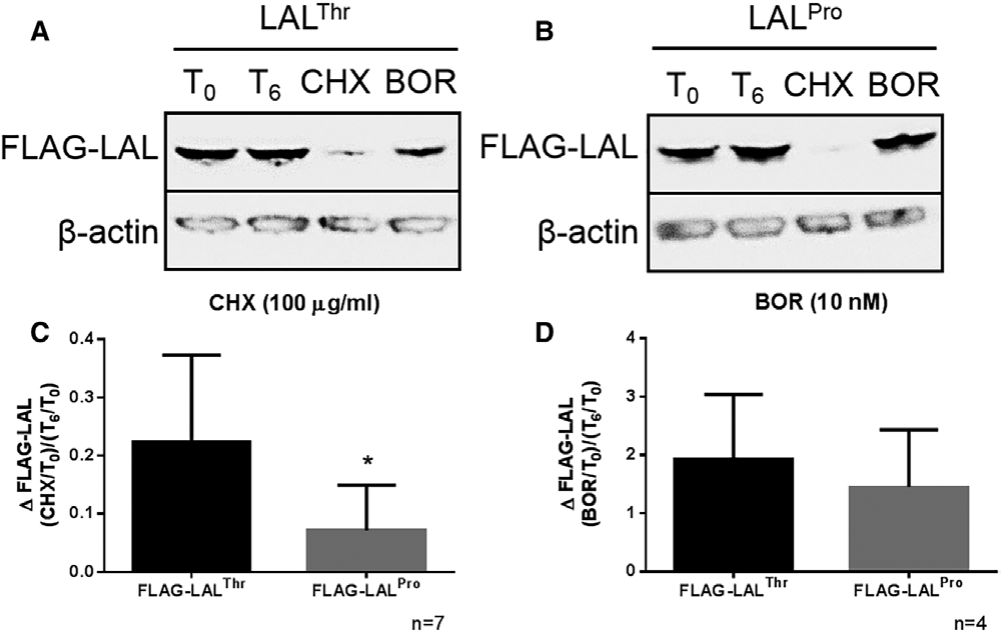
Risk coronary artery disease (CAD)–associated LIPA coding variant (LAL^Pro^) is degraded at an increased rate to nonrisk CAD-associated LIPA coding variant (LAL^Thr^). 1×10^6^ COS7 cells were transfected with 8 μg FLAG-lysosomal acid lipase (LAL) plasmid and cultured for 24 hours before inhibition of either protein synthesis (cycloheximide [CHX] 100 μg/mL) or the proteasome (bortezomib [BOR] 10 nmol/L) for 6 hours and cell lysates prepared. Whole cell lysates were immunoblotted for FLAG-LAL and levels normalized to β-actin, with representative blots shown in **A** and **B**. ΔFLAG-LAL levels in CHX/BOR-treated samples (CHX or BOR/T_0_) were normalized to ΔFLAG-LAL levels in unstimulated samples (T_6_/T_0_), with cumulative CHX data shown in **C** and cumulative BOR data shown in **D**. Data shown are mean±SD, with statistical significance indicated as *P<0.05, unpaired *t* test.

We next investigated whether the difference observed in protein levels from FLAG-LAL^Pro^ and FLAG-LAL^Thr^ transfection was because of a difference in LAL degradation. COS7 cells transfected with FLAG-LAL^Thr^ or FLAG-LAL^Pro^ were incubated for 3 or 6 hours with 100 μg/mL cycloheximide to inhibit protein synthesis, and protein levels were measured by Western blotting. FLAG-LAL^Pro^ was degraded more quickly than FLAG-LAL^Thr^ at 6 hours (*P*=0.035; Figure 3A through 3C; Figure III in the online-only Data Supplement). There was no difference in protein levels between FLAG-LAL^Pro^ and FLAG-LAL^Thr^ after treatment with 10 nM of the proteasome inhibitor bortezomib for 6 hours (*P*=0.547; Figure 3A through 3D).

### Analysis of LAL Expression Levels, Activity, and Function in Macrophages

**Figure 4.**
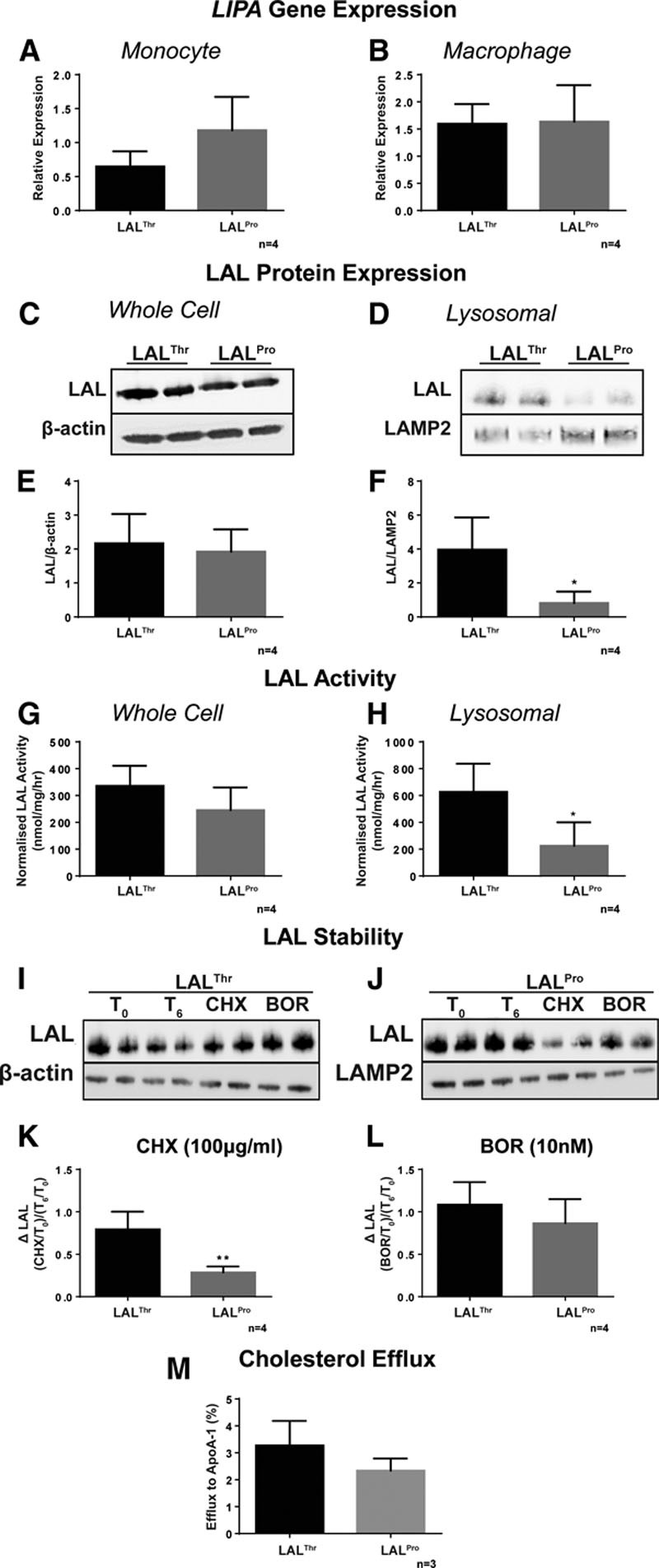
Macrophages from individuals with the risk allele of rs1051338 show reduced lysosomal acid lipase (LAL) activity compared with those with nonrisk allele. PBMC (peripheral blood mononuclear cell) from individuals homozygous for nonrisk coronary artery disease (CAD)–associated LIPA coding variant (LAL^Thr^) or risk CAD-associated LIPA coding variant (LAL^Pro^) were seeded for 2 hours before extensive washing to remove nonadhered cells. Adhered monocytes were either lysed and *LIPA* mRNA expression measured by quantitative reverse transcriptase polymerase chain reaction (qRT-PCR; **A**) or cultured in M-CSF (macrophage colony-stimulating factor; 50 ng/mL) for a further 7 days before *LIPA* mRNA expression quantification (**B**). Whole cell and lysosomal lysates from macrophages cultured from individuals homozygous for LAL^Thr^ or LAL^Pro^ were collected for LAL protein expression, activity, and stability analysis. Whole cell and lysosomal lysates were immunoblotted for LAL, with levels normalized to β-actin or LAMP2 (lysosome-associated membrane protein 2), respectively. Representative whole cell and lysosomal blots are shown in **C** and **D**, with cumulative data shown in **E** and **F**, respectively. LAL activity in both whole cell and lysosomal was quantified using the 4-MUP (4-methylumbelliferyl phosphate) assay and normalized to protein loading control (**G** and **H**, respectively). Protein synthesis or the proteasome were inhibited for 4 hours and cell lysates prepared. Whole cell lysate were immunoblotted for LAL and levels normalized to β-actin, with representative blots shown in **I** and **J**, and ΔLAL cumulative cycloheximide (CHX) and bortezomib (BOR) data shown in **K** and **L**, respectively. Macrophages were loaded with acetylated low-density lipoprotein (LDL; 50 μg/mL) and [^3^H] cholesterol for 30 hours, with efflux to apolipoprotein (apo)A1 measured after 4 hours (**M**). Data shown are mean±SD, with statistical significance indicated as *P<0.05 and **P<0.01, unpaired *t* test.

To investigate whether the findings from the transfection experiments were reflected in a relevant cell type from individuals carrying different genotypes of *LIPA*, circulating monocytes were isolated from healthy adult individuals homozygous for either the risk (n=4) or nonrisk alleles (n=4) of rs1051338 and differentiated into macrophages. *LIPA* expression was measured using quantitative real-time polymerase chain reaction in the isolated monocytes and the derived macrophages. Monocytes with the risk genotype showed a nonsignificant trend toward higher LAL mRNA level compared with nonrisk monocytes (*P*=0.097; Figure 4A). However, there was no difference in *LIPA* mRNA levels in macrophages with the contrasting LIPA genotypes (*P*=0.925; Figure 4B). Consistent with this, there was no genotype-related difference in LAL protein levels in whole macrophages (*P*=0.667; Figure 4C and 4E); however, there was significantly reduced lysosomal LAL protein in risk compared with nonrisk macrophages (*P*=0.02; Figure 4D and 4F). LAL activity was not significantly different between risk and nonrisk genotype in whole cells (*P*=0.164; Figure 4G), but in the lysosomal fraction, LAL activity was significantly reduced in the risk genotype compared with that in nonrisk genotype (*P*=0.026; Figure 4H). The rate of LAL degradation on cycloheximide treatment was significantly greater in macrophages with the risk genotype compared with those with nonrisk genotype (*P*=0.004; Figure 4I through 4K), while no difference in the levels of LAL protein were identified after inhibition of protein degradation (*P*=0.299; Figure 4I through 4L). To examine whether the reduced lysosomal expression and activity of LAL in macrophages from those carrying the risk allele had functional downstream consequences, macrophages from risk and nonrisk genotypes were incubated with the modified lipoprotein acetylated LDL and subsequent cholesterol efflux to apolipoprotein A1 measured. Macrophages from subjects carrying the risk genotype showed a ≈25% reduced efflux capacity compared with those with the nonrisk genotype, although this difference was not significant (*P*=0.187; Figure 4M).

## Discussion

One of the major challenges in understanding the disease-associated loci found through genome-wide association studies is the identification of the causal variant and how it alters the function of the affected gene. In the majority of loci, the disease-associated variants fall in noncoding regions and affect gene expression rather than altering the coding sequence of a gene; however, the 10q23 CAD risk locus is more complex because it is both associated with increased expression of *LIPA* in some cells and tissues and contains a variant that changes the amino acid sequence of the encoded LAL protein. In this study, we investigated the effect of the CAD-associated coding variant, rs1051338, and found that the risk allele caused an increased degradation of LAL, resulting in reduced lysosomal levels of LAL protein and reduced activity. These results are consistent with the early onset atherosclerosis seen with loss of function *LIPA* mutations in CE storage disease^15–21^ and with data from mouse models.^22–26^

The coding variant causes a threonine to proline change within the signal peptide of LAL. Signal peptides are necessary for the correct and efficient targeting of certain proteins to specific organelles, and the CAD-associated variant is in the hydrophobic core of the signal peptide, a region essential for cotranslational translocation into the endoplasmic reticulum by the signal recognition particle.^34^ The α-helical structure and hydrophobicity of the hydrophobic core are known to increase the efficiency of protein secretion.^35,36^ Prolines are known α-helix breakers,^37,38^ and our in silico analysis predicted that the risk allele of rs1051338 disrupts the α-helix, reducing the hydrophobic core, and changes the predicted signal peptide cleavage site. Although these in silico findings indicate a clear alteration in the signal peptide, such findings should be confirmed using structural biology approaches. In addition, several studies have reported that mutations within signal peptide hydrophobic core regions impair translocation and result in protein degradation.^39–42^ Overexpression of plasmids containing either the risk or nonrisk allele of rs1051338 confirmed that LAL^Pro^ had significantly reduced lysosomal protein levels and activity. We also found a trend toward reduced LAL protein levels and activity in both whole cell and extracellular fractions, suggesting that the single nucleotide polymorphism does not result in mistrafficking of LAL. Instead, by treating cells with the protein synthesis inhibitor cycloheximide or the proteasome inhibitor bortezomib, we were able to show that LAL^Pro^ is directed toward the proteasome. Analyses of primary macrophages homozygous for either the risk or nonrisk genotype supported the results of the overexpression system.

We went on to test whether the changes in lysosomal LAL activity had a functional effect on cholesterol homeostasis. In atherosclerosis, modified LDLs are endocytosed in an unregulated process, leading to dramatic increases in intracellular FC concentrations. Excess FC is potentially cytotoxic to cells, so inherent mechanisms limit intracellular FC concentration build-up, including upregulation of cholesterol efflux^43,44^ and re-esterification of FC to form nontoxic, neutral pH, lipid droplets.^45,46^ Lipid droplet degradation was thought to be performed exclusively by cytoplasmic neutral CE hydrolases^47^; however, recent evidence indicates a significant role for lysosomal lipid autophagy for modified LDL metabolism in macrophages.^48,49^ A key role for LAL in this process was highlighted in cholesterol-loaded macrophages, when chemical inhibition of LAL reduced cholesterol efflux to apolipoprotein A1 by >50%.^48,49^ We, therefore, investigated whether rs1051338 genotype affects efflux to apolipoprotein A1 in acetylated LDL-loaded macrophages and found a reduction of ≈25% in efflux to apolipoprotein A1 (*P*=0.187) in macrophages isolated from individuals homozygous for the risk allele. Although not significant, the reduction is in the expected direction; however, interindividual variation means a much larger sample size is required to confirm the functional effects of rs1051338. In LAL deficiency (Wolman disease and CE storage disease), the reduced LAL activity can impair ATP-binding cassette transporter A1 expression in response to LDL loading, leading to reduced cholesterol efflux, an effect that is reversed on treatment with recombinant human LAL.^50^ Indeed, a Phase 3 trial of LAL replacement therapy has recently reported improvements in lipid profile in individuals with LAL deficiency.^51^

Our data suggest that the CAD-associated coding variant results in reduced LAL activity in the lysosomes of macrophages and, therefore, implies that raising LAL levels might be beneficial as a treatment for CAD. However, it is important to emphasize that the effects on LAL activity are complicated by the association between the CAD-risk genotype and *LIPA* expression, which have been identified by multiple studies.^27–30^ Ours is the first study to suggest that the CAD-risk genotype results in a reduction in LAL activity rather than an increase as indicated by gene expression analysis. At present, it is unclear if the association with *LIPA* expression is pertinent to CAD risk or how it relates to the functional effects of the coding variant. The increased *LIPA* expression could be a bystander effect of a high LD variant that falls in a regulatory region or potentially a feedback mechanism caused by the reduced LAL activity induced by the coding variant. In our analysis, we observed a trend toward increased *LIPA* mRNA levels in primary monocytes homozygous for the risk allele but no difference between genotypes in the differentiated macrophages. These results reflect the effects seen in the Cardiogenics consortium’s gene expression analysis of monocytes and macrophages.^9,28^ In monocytes collected from control subjects, the risk genotype (rs2246833) is strongly associated with *LIPA* expression (*P*=6.6×10^−^^64^; β=0.44, n=395) but is attenuated in macrophages (*P*=0.0001; β=0.06, n=305). The difference in effect size between monocytes and macrophages suggests some cell type–specific regulation of *LIPA* expression, and further investigation of the CAD-associated single nucleotide polymorphisms at this locus is required to determine the relevance of *LIPA* expression and to confirm the effects of the coding variant.

Our findings provide a possible explanation for the association between chromosome 10q23 and an increased risk of CAD and is consistent with recent studies describing the role of LAL in the metabolism of modified LDL in macrophages.^48^ However, several limitations of our study and findings need to be highlighted. First, the majority of our findings are from experiments involving overexpression of LAL in COS7 cells and may not accurately reflect the action of the protein processing machinery or LAL activity of a disease-relevant cell type. Second, while our findings in primary macrophages are concordant with those in COS7 cells, the small sample size limited our analysis of the functional consequences of reduced lysosomal LAL activity, and our findings, therefore, require replication in larger cohorts to verify the effects of rs1051338 on lysosomal LAL expression, activity, and cholesterol homeostasis. Finally, we only investigated the coding variant rs1051338 and cannot exclude other high LD variants that affect LAL function and contribute to CAD risk. Indeed, the association between the CAD risk genotype and increased *LIPA* expression has not been studied, and to fully understand the mechanism underlying the disease association will require each of the variants at the locus to be examined individually and in combination. Future work using genome editing could allow the generation of an allelic series comprising the coding variant and other high LD variants in induced pluripotent stem cells prior to differentiation into monocytes and macrophages. Such experiments would identify the relative contribution of each variant to LIPA expression and activity.

In conclusion, our findings suggest that the CAD-associated variant rs1051338, which causes a missense change in the signal peptide of LAL, disrupts the normal sorting and transport of LAL to the lysosome. LAL containing the risk allele is prone to cytosolic proteasomal degradation, reducing lysosomal LAL protein and activity. Understanding how this affects cholesterol metabolism may provide novel therapeutics for CAD through the modulation of macrophage cholesterol concentration.

## Acknowledgments

Many thanks to Paul Helquist and Olaf Wiest from the University of Notre Dame, Indiana, for the academic provision of lalistat-2.

## Sources of Funding

This work was funded by the British Heart Foundation and was also supported by a Transatlantic Networks of Excellence Award (12CVD02) from The Leducq Foundation and the FP7 European Union funded project CVgenes@target (261123). N.J. Samani is a UK National Institute for Health Research Senior Investigator.

## Disclosures

None.

## Supplementary Material

## Highlights

- This study identifies a coding variant in *LIPA* as likely causal variant at the 10q23 coronary artery disease–associated locus.
- The variant increases lysosomal acid lipase protein degradation, causing reduced lysosomal lysosomal acid lipase activity in macrophages.
