# Supplementary figures and images for: Coronary Artery Disease–Associated LIPA Coding Variant rs1051338 Reduces Lysosomal Acid Lipase Levels and Activity in Lysosomes
Source: Arterioscler Thromb Vasc Biol. 2017 May 24;37(6):1050–7. doi: 10.1161/ATVBAHA.116.308734 (PMC5444428; doi:10.1161/ATVBAHA.116.308734)

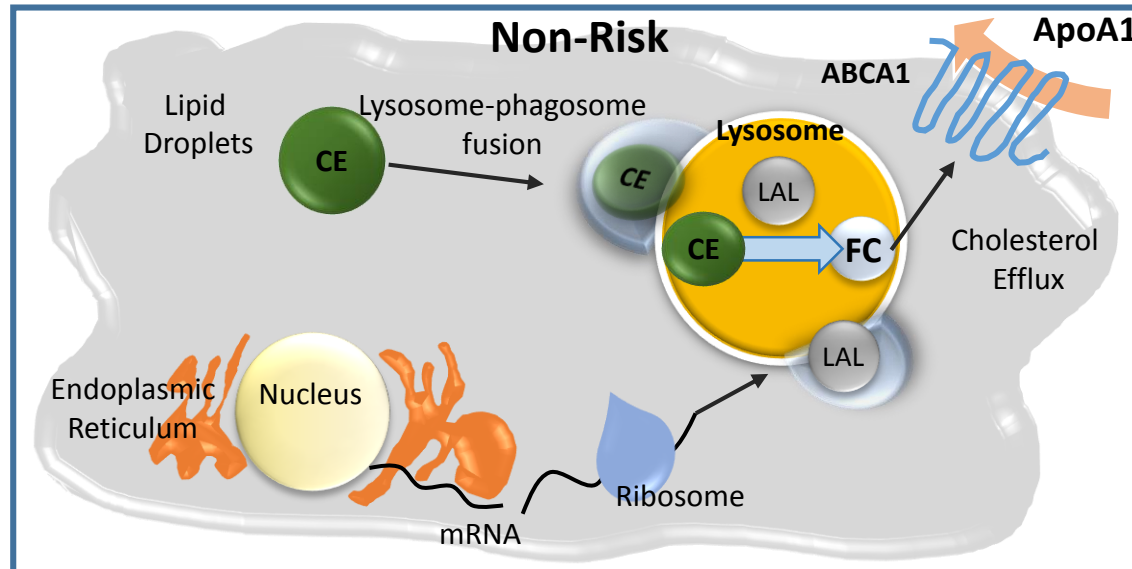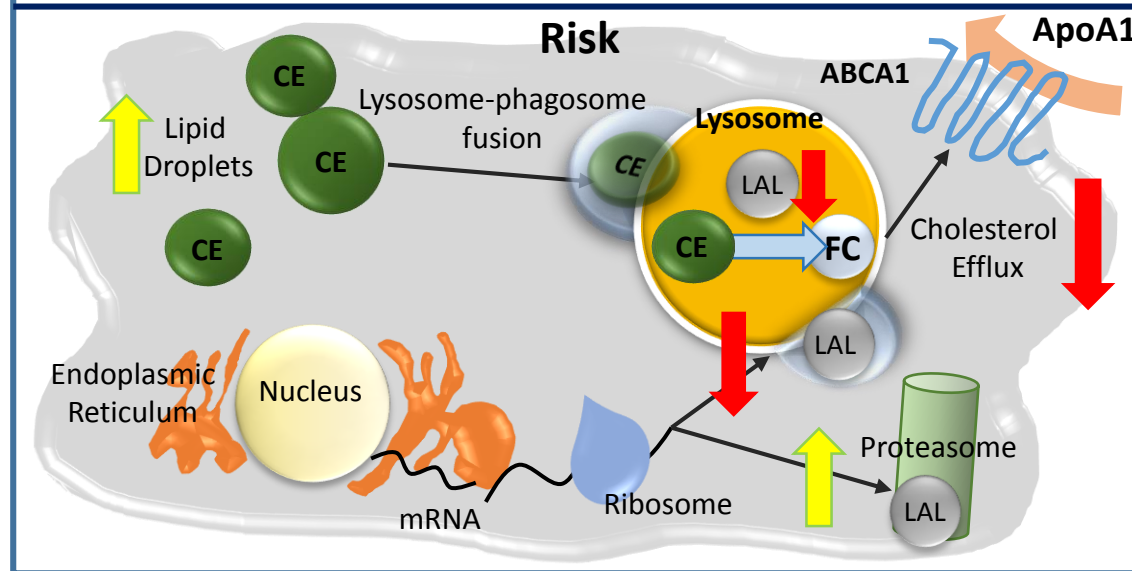

Supplement: Supplementary file 1 [file atv-37-1050-s001.pdf]
