# Supplementary material for: Coronary Artery Disease–Associated LIPA Coding Variant rs1051338 Reduces Lysosomal Acid Lipase Levels and Activity in Lysosomes
Source: Arterioscler Thromb Vasc Biol. 2017 May 24;37(6):1050–7. doi: 10.1161/ATVBAHA.116.308734 (PMC5444428; doi:10.1161/ATVBAHA.116.308734)
Supplement: Supplementary file 2 [file atv-37-1050-s002.pdf]

Material and Methods

Materials

All materials were purchased from Thermo Fisher Scientific (Warrington, U.K.) unless otherwise stated. Bovine serum albumin (BSA), cardiolipin, cycloheximide, and mouse monoclonal anti-FLAG were purchased from Sigma-Aldrich (Dorset, U.K.). Bortezomib was purchased from Merck Millipore (Watford, U.K.). Human macrophage colony stimulating factor (hM-CSF) was from Peprotech (London, U.K.). Mouse monoclonal anti-LIPA, mouse monoclonal anti-LAMP2, and mouse-monoclonal anti-β-actin, were from abcam® (Cambridge, U.K.). Anti-rabbit and anti-mouse horseradish peroxidase (HRP)-conjugated secondary antibodies were from Cell Signalling Technology (Leiden, NL).

Signal Peptide Analysis

Signal peptide *in silico* analyses of the missense variant p.Thr16Pro were performed using several web-based tools: SignalP 4.1[1] identified signal peptidase I cleavage sites, and PSIPRED v3.3[2, 3] was used to predict protein secondary structure.

Generation of LIPA expression plasmids

The full-length *LIPA* open reading frame was amplified by RT-PCR from cDNA homozygous for the non-risk allele using forward primer: 5'-ACCCAAGCTTGGTACCATGAAAATGCGGTTCTTGGGGTT-3' and reverse primer: 5'-AAAATACAGGTTCTCGAGCTGATATTTCCTCATTAGAT-3'. The purified PCR product was cloned into the pLEICS-49 vector, which contains a C-terminal FLAG tag, by The Protein Expression (PROTEX) Laboratory, University of Leicester (Leicester, UK), The risk allele was introduced by site directed mutagenesis using forward primer 5'-TGGTCTGTTTGGTTCTCTGGCCCCTGCATTCTGAGGGGTCT-3' and reverse primer: 5'-AGACCCCTCAGAATGCAGGGGCCAGAGAACCAAACAGACCA-3'.

Cell Culture and Transfection

COS7 cells were cultured in isolation at 37°C in a 5% CO_2 incubator. Cells were maintained in DMEM media containing 10% (*v/v*) foetal calf serum (FCS) and 1% penicillin/streptomycin. Cells were transfected with FLAG-LAL^{Thr} or FLAG-LAL^{Pro} using the NEPA21 Electroporator system (Nepagene, Dublin, IRE). 1x10^6 cells were resuspended with 8 µg FLAG-LAL plasmid (in the presence or absence of 2µg eGFP where stated) in OptiMEM® and transferred to an EC-002 cuvette for electroporation. Transfected cells were cultured for 24 or 48 hours prior to treatment. Transfection efficiency was indirectly determined by calculating the percentage of fluorescent cells within the total number of cells quantified in 4 random fields of view per sample (2 fields of view per duplicate well) using an EVOS™ cell imaging system.

Subject recruitment and identification of rs105338 homozygotes

We recruited 26 healthy Caucasian individuals within the University of Leicester Cardiovascular Science Department (University of Leicester Ethics Reference ahg5-97b2). Whole blood was collected and genomic DNA extracted using the QIAsymphony® DNA kit (Qiagen®). Genotyping for the rs2246942 variant was performed using a TaqMan® SNP genotyping allelic discrimination assay. Of the 26,

4 individuals were found to be homozygous for the risk SNP variant (GG), and 12 were homozygous for the non-risk allele (AA). The 4 homozygous risk individuals were age and gender matched to homozygous non-risk individuals (50:50 male:female for each group, mean age 33yrs (GG) vs 35.25yrs (AA)). In order to confirm that rs2246942 correctly identified individuals for the rs1051338 (r^2=1) signal peptide variant a surrounding 240 bp region of genomic DNA was amplified (forward primer 5'-AATGAAAATGCGGTTCTTGG-3' and reverse primer 5'-GGTTTGGGACCTTTGTCAGA-3') and sequenced.

Human monocyte derived macrophage purification

Human monocyte-derived macrophages were purified from the blood of healthy Caucasian individuals with homozygous genotypes for either the non-risk or risk SNP variant rs1051338, with experiments performed in age and gender matched pairs. The peripheral blood mononuclear cell (PBMC) populations were prepared by centrifugation over Lymphoprep™ (STEMCELL Technologies, Cambridge, U.K.) at 800*g* for 30 minutes. $6x10^6$ PBMCs were seeded per well of a 6 well plate and incubated in a humidified incubator at 37 °C and 5% CO_2 in serum free RPMI-1640 media for 2 hours. Cells were washed extensively, and adhered monocytes were cultured in macrophage-serum free media (M-SFM) supplemented with human macrophage colony stimulating factor (50 ng/mL) for a further 7 days, with media changed on day 3.

LAL Protein Degradation Studies

FLAG-LAL transfected COS7 cells (24 hours post-transfection) or primary macrophages were cultured in the presence of cycloheximide (100 µg/mL) or bortezomib (10 nM) for up to 6 hours to inhibit protein synthesis or the proteasome respectively based on preliminary time course experiments. Unstimulated cell lysates were collected immediately prior to stimulation, and samples were collected from both unstimulated and stimulated cells at either 6 hours or 4 hours post-stimulation (COS7 transfected and primary macrophage samples respectively). Samples were then immunoblotted for LAL and levels normalised to β-actin.

Lysosome Enrichment

Adherent cells were removed using 1x trypsin/ethylenediaminetetraacetic acid (EDTA) solution, pelleted and resuspended in a lysosomal buffer (0.1% BSA, phosphate buffer saline solution containing Halt™ protease inhibitors and 0.5 µM EDTA). Cells were broken up by sonication, with an aliquot of lysate reserved for whole cell lysate analysis. The remaining lysate was centrifuged at 2,000 *g* for 12 minutes at 4^0C to pellet the pre-lysosomal fraction. The lysate was transferred to a fresh tube and centrifuged at 15,000 *g* for 60 minutes to pellet the lysosomal fraction lysate, which was resuspended in lysosomal buffer for analysis.

RNA Studies

Transfection efficiency was directly determined from cDNA levels from cells transfected with 8 µg plasmid with lysates collected after 24 hours: Total RNA was prepared using the RNeasy® Mini kit (Qiagen®, Crawly, U.K.), and genomic DNA removed by DNase I incubation using the RNase-Free DNase Set (Qiagen®). Quantitative real time (qRT-PCR) was conducted from samples using the SYBR®

Green master mix, the forward primer: 5’-TGGCTTGCAGATGTCTACGA-3’ and reverse primer: 5’-TAATCGTGATGGTGATGCGC-3’ (the forward primer bound within *LIPA* and the reverse primer binding within FLAG to ensure amplified products were of transfected plasmid origin). LIPA expression levels were quantified in monocytes and macrophages using the forward primer 5’-CTTGCTGATGCTGGTTTTGA-3’ and reverse primer 5’- GCCTTGAGAATGACCCA CAT-3’. Amplification was carried out in triplicate using a Rotor-Gene® Q (Qiagen®). Samples were normalised to the 36B4 housekeeping gene using the forward primer: 5’-TCGACAATGGCAGCATCTAC-3’ and reverse primer: 5’-GCCTTGACCTTTTCAGCAAG-3’.

Western Blot Analysis

Protein content was measured using the Novex® protein separation kit. Equal amounts of protein lysates were separated by SDS-PAGE before blotting onto nitrocellulose membrane. Membranes were probed with anti-FLAG, anti-LAL, anti-β-actin, or anti-LAMP2 primary antibodies, detected with horseradish peroxidase-conjugated secondary antibodies and visualised by enhanced chemiluminescence (GE Healthcare, Amersham, U.K.). Quantitative signals were derived by densiometric analysis using ImageQuant™ TL on an ImageQuant™ LAS 4000 Luminescent Image Analyzer (Fujifilm, Dusseldorf, Germany) with protein content corrected to β-actin or LAMP2 protein levels for whole cell or lysosomal fractions respectively. Conditioned media protein content was concentrated before SDS-PAGE: media was centrifuged at 15,000 *g* for 5 minutes to pellet cellular debris. An equal volume of 20% Trichloroacetic acid (TCA) was added to media and samples were left on ice for 30 minutes. Samples were centrifuged at 15,000 *g* for 15 minutes at 4^0C before supernatant was removed and acetone added to the resulting pellet. Pellets were dried at 95^0C for 10 minutes before resuspended in SDS-sample buffer.

LAL activity

LAL activity in transfected COS7 cells and macrophages were measured using the *ex vivo* 4-methyl-umbelliferone-palmitate (4-MUP) fluorometric lipase enzyme assay[4, 5]. Whole cell, lysosomal lysates, or conditioned media were incubated in duplicate in the absence or presence of the selective LAL inhibitor lalistat-2 (gift of Prof. Helquist and Prof. Wiest, University of Notre Dame, Indiana, U.S.A.) in a 4-MUP substrate buffer solution (0.4:1:14; 4-MUP:cardiolipin:100 mM Sodium Acetate buffer, pH 4) for 4 hours at 37^0C in the dark. The reaction was stopped by addition of 150 mM EDTA, pH 11.5 solution. Fluorescence intensity was measured using a fluorometer (Ex. 340 nm/Em. 510 nm) (NOVOstar, BMG Labtech Ltd., Aylesbury, U.K.). Enzyme activity was reported as nmol/mg of protein/hour, normalised to whole cell (β-actin) or lysosome (LAMP2) protein levels from the same sample.

Cholesterol Efflux

Macrophages were loaded for 30 hours with 50 µg/ml acetylated LDL that had been pre-incubated with [^{3}H] labelled cholesterol (5 µCi/ml) in M-SFM supplemented with 1% FCS and 0.5% gentamycin (250 µg/ml). Labelling media was removed and cells were equilibrated for 16 hours in M-SFM supplemented with 0.2% FCS and cAMP (300 µM). Cholesterol efflux was determined in the presence of Apolipoprotein A1 (ApoA-1) (50 µg/ml) for 4 hours at 37°C in efflux media (serum-free Minimum Eagles Media supplemented with 14 mM HEPES and 150 µM cAMP). The amount of

cholesterol released by non-efflux pathways was controlled for by parallel incubation of cells in the absence of Apo-A1. After efflux, media was collected and cellular debris removed by centrifugation. To assess uptake of [^{3}H] cholesterol cells were lysed in 0.2N sodium hydroxide with 0.1% SDS. Radioactivity was determined by liquid scintillation counting (Tri-Carb® 2910 TR Liquid Scintillation Analyzer, PerkinElmer, Beaconsfield, U.K.). Cholesterol efflux percentage was calculated as: 100 x ([^{3}H] count in presence of ApoA-1 - [^{3}H] count in absence of ApoA-1) / cellular [^{3}H] count.

Statistical Analyses

Data are presented for cells cultured on at least four separate occasions and are expressed as mean ± standard deviation (SD). Data were analysed (GraphPad Prism, San Diego, CA) using an unpaired T-test.

References

1. Petersen TN, Brunak S, von Heijne G, Nielsen H. Signalp 4.0: Discriminating signal peptides from transmembrane regions. *Nature methods*. 2011;8:785-786
2. Buchan DW, Minneci F, Nugent TC, Bryson K, Jones DT. Scalable web services for the psipred protein analysis workbench. *Nucleic acids research*. 2013;41:W349-357
3. Jones DT. Protein secondary structure prediction based on position-specific scoring matrices. *Journal of molecular biology*. 1999;292:195-202
4. Dairaku T, Iwamoto T, Nishimura M, Endo M, Ohashi T, Eto Y. A practical fluorometric assay method to measure lysosomal acid lipase activity in dried blood spots for the screening of cholesteryl ester storage disease and wolman disease. *Molecular genetics and metabolism*. 2014;111:193-196
5. Hamilton J, Jones I, Srivastava R, Galloway P. A new method for the measurement of lysosomal acid lipase in dried blood spots using the inhibitor lalistat 2. *Clinica chimica acta; international journal of clinical chemistry*. 2012;413:1207-1210
